# DEVELOPMENT AND VALIDATION OF A RISK PREDICTION MODEL FOR SARCOPENIA IN COMMUNITY-DWELLING OLDER ADULTS

**DOI:** 10.1093/geroni/igad104.2791

**Published:** 2023-12-21

## Abstract

Reliable identification of older populations at high risk of sarcopenia is essential for initiating targeted preventive measures and follow-up, but no sarcopenia risk prediction model is available. Our objective was to develop and validate a prediction model for calculating the 1-year absolute risk of sarcopenia occurrence for older people in the community. The analytic samples for the prediction model derivation were conducted on data from one prospective population-based cohort. The main outcome was the development of sarcopenia within 1 year (defined by 2019 Asian Working Group for Sarcopenia consensus). Multivariate logistic regression analysis was conducted to examine those independent risk factors for sarcopenia and the sarcopenia risk-predicting model was developed using the R software. In the development cohort, 1042 non-sarcopenia residents were enrolled, of whom the mean age was 66.6±5.0 years, 631 participants were women, and 87 participants developed sarcopenia at the 12-month follow-up. The final model contains 7 risk factors-age, gender, BMI, low physical activity, malnutrition, pain, and calf circumference. The model showed excellent discrimination (AUROC 87%, 95% CI 0.83–0.90), calibration capability (slope 0.93) and clinical utility. Internal validation was conducted using 200 times bootstrapping technique and showed a stable discriminative ability (corrected AUROC of 0.85). High risk thresholds of 10% or more were able to identify high-risk individuals of sarcopenia, and low risk thresholds less than 2.5% were useful to rule out the likelihood of sarcopenia. In summary, the sarcopenia risk-predicting model for community-dwelling older people consists of 7 readily available predictors and has an excellent predictive ability.

